# The socially weighted encoding of spoken words: a dual-route approach to speech perception

**DOI:** 10.3389/fpsyg.2013.01015

**Published:** 2014-01-09

**Authors:** Meghan Sumner, Seung Kyung Kim, Ed King, Kevin B. McGowan

**Affiliations:** Department of Linguistics, Stanford UniversityStanford, CA, USA

**Keywords:** speech perception, spoken word recognition phonetic variation, episodic lexical access, social weighting

## Abstract

Spoken words are highly variable. A single word may never be uttered the same way twice. As listeners, we regularly encounter speakers of different ages, genders, and accents, increasing the amount of variation we face. How listeners understand spoken words as quickly and adeptly as they do despite this variation remains an issue central to linguistic theory. We propose that learned acoustic patterns are mapped simultaneously to linguistic representations and to social representations. In doing so, we illuminate a paradox that results in the literature from, we argue, the focus on representations and the peripheral treatment of word-level phonetic variation. We consider phonetic variation more fully and highlight a growing body of work that is problematic for current theory: words with different pronunciation variants are recognized equally well in immediate processing tasks, while an atypical, infrequent, but socially idealized form is remembered better in the long-term. We suggest that the perception of spoken words is socially weighted, resulting in sparse, but high-resolution clusters of socially idealized episodes that are robust in immediate processing and are more strongly encoded, predicting memory inequality. Our proposal includes a dual-route approach to speech perception in which listeners map acoustic patterns in speech to linguistic and social representations in tandem. This approach makes novel predictions about the extraction of information from the speech signal, and provides a framework with which we can ask new questions. We propose that language comprehension, broadly, results from the integration of both linguistic and social information.

## INTRODUCTION

Spoken words are highly variable. A single word may never be uttered the same way twice. As listeners, we regularly encounter speakers of different ages, genders, and accents, increasing the amount of variation we face. How listeners understand spoken words as quickly and adeptly as they do despite this variation remains an issue central to linguistic theory. While variation is often couched as a *problem*, we go through our daily lives with relatively few communicative breakdowns. In our perspective, variation is *key* to explaining how listeners understand spoken words uttered at various speaking rates and styles by various speakers, each with their own idiolect, each a member of a broader dialect. We propose that learned acoustic patterns are mapped simultaneously to linguistic representations and to social representations and suggest that listeners use this variation-cued information and encode speech signals directly to both linguistic and social representations in tandem. Our approach includes the traditional route of encoding of speech to linguistic representations and a proposed second route by which listeners encode acoustic patterns to social representations (e.g., the acoustic cues that constitute *clear speech* are stored as sound patterns independent of the lexicon). This second route provides a mechanism for what we call *socially weighted encoding*. Social weighting enables infrequent, but socially salient tokens to result in robust representations, despite being less often experienced compared to highly frequent tokens. Social weighting explains a variety of effects of the recognition and recall of spoken words that are not easily accounted for in current models that rely heavily on raw token frequency (often estimated from corpus counts). We present a hypothesis that considers *linguistic* *experience* from a listener’s perspective as both a quantitative and a qualitative measure.

In this paper, we examine a body of literature that has investigated the perception and recognition of words with different pronunciation variants (e.g., center produced with or without a word-medial [t]; city produced with a word-medial tap, 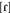, or with a [t]). We highlight a paradox that arises from the focus on representations (as opposed to mechanisms that build and access those representations) and from the peripheral treatment of word-level phonetics (c.f., Keating, 1998). In doing so, we illuminate some data that are difficult for current theory to handle. First, all pronunciation variants are recognized equally well by listeners in immediate recognition tasks in spite of the huge difference in observed rates of variant frequency (which we call *recognition equivalence*). And, words pronounced with infrequent, but *idealized forms*^1^ To account for both recognition equivalence and memory inequality, we not only distinguish atypical forms from typical forms, but also distinguish different atypical forms. This distinction is necessary since *idealized* atypical forms are remembered better than non-idealized forms (whether typical or atypical). To do this, we present a novel view of how lexical representations are built and accessed from both quantitative and qualitative experience. Specifically, we propose that socially salient tokens are encoded with greater strength (via increased attention to the stimulus) than both typical and atypical non-salient tokens (which we call *social-weighting*). Our approach suggests that a representation derived from one instance of a strongly encoded socially salient token may be as robust as one derived from a high number of less salient, default tokens. Much work in speech perception has investigated the many-to-one mapping of variable signals to a single linguistic representation. We instead pursue a one-to-many approach in which a single speech string is mapped to multiple social and linguistic representations. We view speech as a multi-faceted information source and pursue a perspective in which language comprehension results from the interactive contributions of both social and linguistic information.

## LISTENER SENSITIVITY TO PHONETIC VARIATION DURING PERCEPTION AND RECOGNITION

Over 20 years ago, auditory memory for acoustic events was found to be highly detailed (Schacter and Church, 1992; Nygaard et al., 1994; Green et al., 1997; Nygaard and Pisoni, 1998; Bradlow et al., 1999). Church and Schacter (1994), for example, investigated implicit memory for spoken words with a series of five priming experiments and found that listeners retain detailed acoustic cues to intonation contour, emotional prosody, and fundamental frequency. However, listeners do not retain detailed memory of amplitude differences suggesting that auditory memory for speech is both highly detailed and selective. This finding, along with years of experimental support, shifted the perspective of the field, moving away from the long-held assumption that phonetic variation in speech is redundant noise that is filtered out as the speech signal is mapped to higher-level linguistic units. Instead, variation was found to be integral to lexical representations and access to those representations. The years of research examining episodic lexicons also led to the emergence of a highly productive research area investigating the effects of *phonetically cued social variation* in speech perception (see Drager, 2010 and Thomas, 2002 for reviews). Phonetically cued social variation refers to those acoustic properties in speech that cue attributes about a talker (e.g., age, gender, accent, dialect, emotional state, intelligence) or a social situation (e.g., careful vs. casual speech style). Listeners use perceived social characteristics of a speaker to guide the mapping of acoustic signals to lexical items (Niedzielski, 1999; Strand, 1999, 2000; Hay et al., 2006a,b; Babel, 2009; Staum Casasanto, 2009; Hay and Drager, 2010; Munson, 2011). When social characteristics and the acoustic input are misaligned, processing can be slowed (Koops et al., 2008) or impaired (Rubin, 1992). When the cued social characteristic is aligned with the speech signal, however, mapping of the acoustic signal to lexical representations can be enhanced (McGowan, 2011; Szakay et al., 2012). This literature has established that memory for spoken words is highly specific and that linguistic representations are built from detailed instances of spoken words.

One consequence of storing specific instances (or *episodes*) of words is that listeners do not store a single representation per lexical item. Instead, a lexical representation arises from the clustering – in some multi-dimensional acoustic space – of a listener’s experiences corresponding to a particular lexical item. Two prominent mechanisms explaining lexical access to clustered episodes have been proposed (Goldinger, 1996, 1998; Johnson, 1997, 2006). While the mechanisms differ slightly, they are both based on a similar principle: when exposed to a speech signal, individual stored episodes are differentially activated as a function of acoustic similarity to the incoming speech signal, and a lexical representation is chosen based on the amount of activation received by each of its component episodes. In both cases, access between the incoming speech signal and word-level representations is direct. Direct access to episodic lexical representations has been supported by a large body of work. Knowledge of a particular speaker’s voice can improve recognition of novel words (Nygaard and Pisoni, 1998), with particular acoustic cues showing differential weighting when used to access lexical representations (Bradlow et al., 1999; Nygaard et al., 2000). Cross-linguistic differences like the classic difficulty of native Japanese speakers with the English /r/-/l/ distinction (long attributed to native phoneme inventory, e.g., Best et al., 2001) are not evident in a speeded recognition task that forces discrimination to be more psychoacoustic. The expected differences emerge when listeners have sufficient time to compare the input acoustic signal directly to the lexicon (Johnson, 2004, Johnson and Babel, 2010). Finally, the literature on phonetically cued social variation presumes direct lexical access (e.g., Munson, 2010). Strand (2000), for example, found that voices that are more stereotypically male (or female) are repeated faster than less stereotypical voices.

The direct mapping of speech to lexical representations is not the only mechanism at work; listeners also map speech to smaller, sub-lexical linguistic units. Subcategorical mismatches in fine phonetic detail have long been known to slow listeners’ phonetic judgments even when ultimate categorical outcomes remain constant (Whalen, 1984, 1989). Listeners use talker-specific distributional properties to shift the category boundaries of pre-lexical (phoneme-like) categories and, crucially, can generalize these across the lexicon (see Sumner, 2011 and Cutler et al., 2010, respectively). The language of discourse can shift listeners’ ability to discriminate vowel category boundaries in the perception of individual words. For example, in a vowel categorization task, native Swedish listeners with high English proficiency more reliably identified vowels along a *set-sat* continuum when the instructions of the task were in their native Swedish than in English (Schulman, 1983). Furthermore, listeners shift phoneme categorization boundaries when there is segmental acoustic evidence pointing to coarticulation (Mann, 1980; Mann and Repp, 1981; Holt et al., 2000). And, listeners use this evidence of coarticulation as soon as it becomes available in the speech signal (Lahiri and Marslen-Wilson, 1991; Ohala and Ohala, 1995; Beddor et al., 2013).

Across studies, evidence has mounted supporting the view that listeners map speech to lexical representations directly, *and* by way of smaller sub-lexical chunks. These and other findings prompted McLennan et al. (2003) to posit a hybrid model of lexical access by which both lexical and sub-lexical chunks are central to the speech perception process (see also Goslin et al., 2012 for additional support). We consider both direct and mediated lexical access to be supported by various lines of research, though our approach does not depend on this distinction. We take this body of work as evidence that listeners are highly sensitive to subtle acoustic variation in speech and that this variation influences linguistic representations. Both mediated and direct access models share the view of phonetic variation as a cue to linguistic representations (that may or may not, in turn, activate social representations). We suggest here that it is equally important to consider the social meaning conveyed by phonetic variation independent of linguistic representations to explain how listeners understand spoken words. In this view, phonetic variation cues sounds, words, speaker attributes, and situational information, and the interpretation of these together results in spoken language understanding.

## PHONETIC VARIATION, RECOGNITION EQUIVALENCE, AND MEMORY INEQUALITY

Listeners hear numerous instantiations of a word and need to understand those variable forms as one word and not another. That is, listeners must map variable *tokens* of a single word *type* to that *type*. This is not a trivial task, as minimal phonetic differences often cue different lexical items. This issue of many-to-one mapping has been traditionally approached in an *either/or* fashion: acoustic tokens either map to specific or abstract representations (though see McLennan et al., 2003 for an alternative approach). This either/or perspective has resulted in a literature that is full of paradoxical results.

Consider /t/-reduction processes in American English (AE). The word *petal* usually sounds like the word *pedal*. In fact, words like these are found to be pronounced with a word-medial tap, 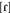, 97% of the time (Patterson and Connine, 2001; Tucker, 2011). Independent of what we think we say, we rarely pronounce a [t] in these words. The 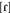/[t] pair is a *pronunciation variant pair* where two sounds may be uttered in the same phonological context: one a phonetically casual production with the frequent 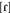, and the other a phonetically careful production with the rare [t]. Other pronunciation variant pairs (or sets) exist in AE. A word like *center* is typically produced sounding like *sen-ner* rather than *sen-ter* (occurring *without* a [t] in all 53 out of the 53 instances in the Buckeye Corpus; Pitt, 2009), and a word like *flute* is typically produced *without* an audible final [t]-release^2^ (see Sumner and Samuel, 2005).

Collapsing across studies that investigate the recognition of words with different pronunciation variants leads to the *representation paradox* (Sumner et al., 2013). This paradox is best illustrated by two conceptually identical studies that examine the perception of words with medial /t/. On one hand, investigating the perception of words pronounced with medial [t] versus medial 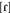 (e.g., bai[t]ing vs. bai
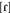
ing), Connine (2004) found that listeners identify tokens as words (rather than non-words) more often when the tokens contained 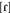, the more frequent variant, as opposed to [t], the infrequent, idealized variant. This finding is similar to other work showing a benefit for the more typical form (e.g., Nygaard et al., 2000). On the other hand, Pitt (2009) investigated the perception of words with or without a post-nasal [t] (e.g., *center* produced as cen[t]er vs. cen[_]er), and found that listeners recognized tokens as words more often when the tokens contained the infrequent [t] instead of the more frequent [n_]. This finding is consistent with other work showing a benefit for the canonical, or what we refer to as an idealized, form (e.g., Andruski et al., 1994; Gaskell and Marslen-Wilson, 1996). The paradox is that these two conceptually identical studies (and numerous other similar studies) show seemingly contradictory results: both frequent non-idealized forms *and* infrequent idealized forms show processing benefits over the other forms.

This body of literature typically investigates effects of words with different pronunciation variants independent of subtle but significant word-level phonetic patterns that co-vary with each variant (see also Mitterer and McQueen, 2009). As discussed in Section “Listener sensitivity to phonetic variation during perception and recognition”, it is well established that listeners are highly sensitive to subtle fluctuations in speech (e.g., McMurray and Aslin, 2005; Clayards et al., 2008; McMurray et al., 2009). To illustrate why the consideration of word-level phonetic variation is important, we again focus on two conceptually similar studies. First, Andruski et al. (1994) investigated the semantic priming of targets by primes beginning with voiceless aspirated stops (e.g., cat–DOG). They found that target recognition was facilitated by semantically related primes beginning with fully aspirated voiceless stops, but not by those beginning with slightly aspirated stops, even though the reduced-aspiration variant is more typical of natural speech. In this case, the pronunciation variant pair (fully aspirated vs. slightly-aspirated voiceless stops) was investigated without consideration of the overall phonetic composition of the word: the slightly aspirated tokens were created by digitally removing the mid-portion of the aspiration from the carefully uttered fully aspirated tokens. This created a slightly aspirated variant with otherwise carefully articulated phonetic patterns (e.g., unreduced vowels, longer segment durations) – a pairing that would likely result in a *voiced* percept to AE ears (Ganong, 1980; Sumner et al., 2013). And, as low-level phonetic mismatches are costly in perceptual tasks (see Marslen-Wilson and Warren, 1994), the benefit for the idealized variant may not be due to access to an idealized representation, but a cost associated with the mismatched form; warranting an alternate explanation.

Sumner and Samuel (2005) used a semantic priming paradigm (similar to Andruski et al., 1994) to investigate the effects of word-final /t/ variation on spoken word recognition. They investigated the recognition of targets (e.g., music) preceded by semantically related (e.g., flute) or unrelated (e.g., mash) prime words. The related primes included words produced with a fully released [t], a coarticulated unreleased 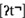, a glottal stop 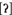, and an arbitrary variant (different from /t/ by a single feature, like [s] in *floose*). Crucially, all variants were naturally uttered and contained typically co-present word-level cues (e.g., vowel glottalization), instead of excised or spliced stimuli. In contrast to Andruski et al. (1994); Sumner and Samuel (2005) found that all word productions (except for the arbitrary variant) were equally able to facilitate the recognition of semantically related targets. Both studies also varied interstimulus intervals, but with different outcomes. Andruski et al. (1994) found a cost for the phonetically incongruent slightly aspirated stops at short ISIs, but not at long ones. Sumner and Samuel (2005) found equivalence across variants at both short and long ISIs. This might suggest that the cost for the more typical, slightly aspirated variant along with the benefit for the fully aspirated variant reported by Andruski et al. (1994) stemmed either from a phonetic mismatch as explained above, or from the comparison between an intact word form and a manipulated one.

Sumner (2013) went one step further and argued that the benefit of idealized forms in studies that compare an infrequent, ideal variant in a careful word-frame to a frequent, non-ideal variant in the same careful word frame is somewhat artificial. She examined the recognition of spoken words with a medial /nt/ sequence, like *splinter*. In a semantic-priming task, words produced with a [t] (e.g., [nt], splin[t]er, the infrequent ideal forms) and words produced without a [t] (e.g., [n_], splin_er, the frequent non-ideal forms) are both *equally* able to facilitate recognition to a semantically related target (e.g., wood) when they were housed in appropriate word frames. Critically, a cost only arises when the frequent [n_] variant is housed in an incongruent carefully articulated phonetic word frame. Similar asymmetries arise in studies that investigate the perception and recognition of assimilated variants depending on the consideration of phonetic variation. For example, Gaskell and Marslen-Wilson (1993) found that listeners recognize a pseudoword like *wickib* as the word *wicked* when produced before a word that begins with a labial (an assimilating context). They attributed this effect to listeners’ dependence on the following context to interpret the underlying sound of a word. But, one could argue that by producing *wickib* with a [b] instead of a naturally assimilated token, critical coarticulatory information is eliminated from the speech signal, forcing listeners to depend on context. Gow (2001, 2002), using a sentential-form priming paradigm, showed that naturally assimilated nasals (those that include residual phonetic cues to the coronal place of articulation) are processed unambiguously as the intended word (e.g., the labial-assimilated /n/ in “*green* beans” is not identical to [m] and the word is not perceived as [grim]). Even more interesting, this was true even when the assimilation-inducing following phonological context was not presented to listeners (Gow, 2003).

McLennan et al. (2003) also used naturally uttered spoken words with medial-t and found that listeners recognize words pronounced with [t] and words pronounced with 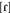 on par with each other. This literature highlights the role of phonetic variation in spoken word recognition but also illuminates a theoretical quandary: when naturally produced, word forms with vastly different token frequencies are all recognized equally well in immediate processing tasks. Muddying the picture even more, Sumner and Kataoka (2013) found, for a monolingual AE listening population, that rhotic AE primes facilitate recognition to semantically related targets (e.g., slend-er–THIN). They also replicated an earlier finding for this population that non-rhotic primes produced by speakers with a New York City (NYC) accent do not facilitate recognition to these targets (e.g., slend-uh–THIN). Critically, though, words that ended in the same non-rhotic variant did facilitate recognition to semantically related target when produced by non-rhotic British English (BE) speakers. In this case, words uttered by an out-of-accent speaker were recognized on par with those produced by a within-accent speaker. These studies illuminate what we call *recognition equivalence*.^3^ In the extreme case reported by Sumner and Kataoka (2013), one might expect differences in the recognition of words that derive from two different out-of-accent talkers, and we might even be able to suggest that differences in quantitative exposure predict the NY – BE split. But, any measure of frequency would include great differences in exposure to productions uttered by a within-accent speaker (AE) compared to an out-of-accent speaker (BE). This equivalence, along with those described above, illuminate the limits of the explanatory power of quantitative frequency measures, and suggest to us that a qualitative measure need also be considered.

In tandem with recognition equivalence is an associated finding that words with infrequent, but idealized variants are remembered better than words with frequent, non-idealized variants. In general, equivalence is much less likely in long-term studies. We call this *memory inequality*. Sumner and Samuel (2005) investigated the effects of word-final /t/ variants on long-term implicit and explicit recognition tasks. The basic design of an implicit (reaction-time based long-term repetition) or an explicit (old/new recognition) task involves presenting listeners with an initial study list and measuring performance on words repeated on a second test list presented 10–20 min later. They found that the performance on the second presentation showed a memory benefit for the idealized [t] variant in both types of tasks. That is, listeners remembered words that were initially presented with a released stop better than those that were initially presented with either an unreleased glottalized stop or a glottal stop. Note that there was no hint of abstraction, in which case high rate of false alarms for words initially presented with other variants should have resulted (see, however, McLennan and Luce, 2005 for arguments in favor of abstraction, though in a much shorter time frame). Instead, listeners had highly detailed memory for words with the infrequent ideal forms.

One possible explanation for memory inequality is that words with final-released [t] are acoustically more salient than their glottalized unreleased or glottal stop counterparts. This type of acoustic salience explanation might predict that words with final-released [t] are encoded more strongly than words with the other two variants. Another option is that the two variants with glottalized vowels made the released version more contextually salient, and therefore, remembered better on second presentation. At first glance, both seem feasible, but follow-up studies have made these unlikely. First, Sumner and Samuel (2009) investigated the effects of cross-accent variants. The particular experiment relevant to the current discussion is a long-term form priming task that examined the recognition of words ending in either a rhotic (*slend-er*) or non-rhotic (*slend-uh*) pronunciation variant. They investigated three listener populations: a group of AE listeners less familiar with the non-rhotic pronunciation, a group of rhotic speakers who were born and raised in the non-rhotic NYC dialect region (Covert-NY), and a group of non-rhotic speakers who were born and raised in the non-rhotic NYC dialect region (Overt-NY). Unsurprisingly, in this long-term memory-based task, AE and Covert-NYC listeners recognized their within-accent rhotic variant with greater speed and accuracy than the out-of-accent, less familiar non-rhotic variant. What was surprising, though, is that Overt-NYC listeners also showed better memory for the AE forms. While an acoustic salience account (either inherent to a sound or created by contextual comparisons) might be supported in the case of final release [t], there is little motivation to suggest that a rhotic vowel is more salient than a non-rhotic vowel, especially when the same pattern holds across listener populations.

A theory that depends heavily on a quantitative measure of frequency will have difficulty with this asymmetry. On one hand, all pronunciation variants are recognized equally well – quickly enough to promote associative spread throughout the lexicon. On the other hand, an atypical pronunciation of a word is remembered better than more typical pronunciations. Reconciling these findings via the notion of abstract representations will not fare well: in such theories, variant pronunciations should generalize to a single abstract form over time, leading to more false attributions of the ideal form in the long-term. This prediction runs counter to years of research showing highly specific memory for linguistic events. A purely frequency-based account faces issues of a different sort, as recognition equivalence is difficult to capture in theories that depend on global production rate (either cross a language or speaker group) as a predictor of lexical access. We propose that the resolution will come from understanding how different word forms are encoded in the first place, and how clusters of representations stemming from differentially encoded spoken words are composed.

## TYPICALITY, FREQUENCY, AND THE ASYMMETRICAL ENCODING OF SPOKEN WORDS

To predict both recognition equivalence and memory inequality from a representation-based perspective, three conditions must hold. First, we need to differentiate between typical and atypical tokens. Second, we need to capture differences between different atypical productions. For example, not all atypical tokens are remembered better than typical tokens. Rather, only atypical, but idealized tokens are remembered better than non-ideal (whether typical or atypical) tokens. Finally, we need to understand how tokens that best match infrequently experienced token clusters can be recognized on par with tokens that best match densely populated, frequently experienced token clusters. For the first condition, the traditional notion of frequency as a measure of typicality is reasonable. This enables us to relate our proposal to past work and to build on current theory. For the second condition, we propose that socially salient tokens are encoded with greater strength (via increased attention to the stimulus) than both typical and atypical non-salient tokens. For the third condition, we suggest that cluster robustness and acoustic overlap with typical clusters account for equivalence.

**FIGURE 1:**
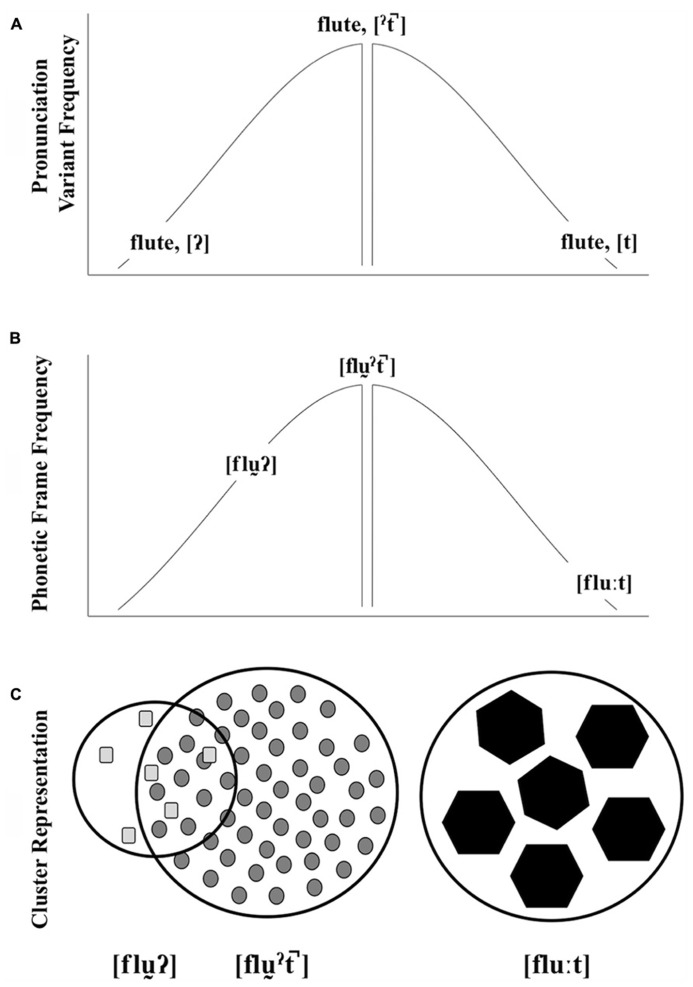
**A schematic illustration of pronunciation variant frequency **(A)**, **phonetic frame** frequency **(B)**, and cluster representation **(C)** for words ending in three t-final variants (used here as symbolic of other kinds of variation).** As depicted, the frequency of two atypical pronunciation variants may be the same, but their relationship to the typical production may differ in terms of word-level phonetic variation. In our schematic, cluster representations, shape corresponds to pronunciation variant, cluster size corresponds to cluster robustness, and token size corresponds to encoding strength. The overlap between the atypical glottal-final form and the typical glottalized, unreleased-final form represent the typicality benefit applied to the atypical form.

Our view of the interactions between pronunciation variant frequency, word-level phonetic frame frequency, and encoding strength is illustrated in **Figure 1**. The top and middle graphics depict the frequency of different pronunciation variants (A: 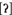, 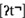,[t]) and phonetic frames (B: [fl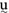], [fl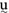], [flu:]) for a particular lexical item (e.g., *flute*). The center of each distribution shows the corresponding typical pronunciation variant or phonetic frame. In this view, a word with an atypical variant may or may not be acoustically similar to a word with a typical variant. For example, the acoustic realization of a glottal-final *word* is similar to the typical production (e.g., glottalized vowel, vowel length, weak/absent release, etc.,). Episodic theories of lexical access provide an insight into how these word forms result in the activation of a particular lexical item. Johnson (2006) has found that words produced in typical female voices are recognized more quickly than words produced in atypical female voices. This typicality benefit arises because lexical access is acoustically mediated: a speech string activates acoustically similar episodes. The phonetic composition of a word produced by a typical female voice would be acoustically similar to the densely populated center of the distribution and a high-level of activation ensues. The phonetic composition of a word produced by a less typical voice maps to a sparse cluster and activation is delayed. This typicality benefit found by Johnson provides a straightforward explanation of how prototype effects emerge in speech processing (Pierrehumbert, 2001). For example, one need not have heard a *particular* typical female voice in order for the voice to benefit from the resonant activation of acoustically similar episodes. The gap depicted in **Figure 1** (A,B) symbolizes the midpoint of the distribution that might represent a prototype gap.

Shifting back to words with final-/t/, like *flute*, the prototype of this lexical item is some production of the word with an unreleased 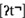 (**Figure 1A**), with a corresponding casually articulated phonetic frame (**Figure 1B**). Atypical productions with acoustic values similar to the center of the distribution benefit from the activation of typical episodes. This is expected for the atypical glottal-final form. Though it differs from the typical form by pronunciation variant of the final sound, there is acoustic overlap with the typical cluster at the word-level (**Figure 1C**). The socially idealized form [flu:t], is atypical in two ways, though. First, the pronunciation variant is atypical. Second, the phonetic composition of the word is also atypical, as [t] typically co-varies with careful speech patterns. In neither case will this form benefit as much from this frequency-based similarity activation as a prototype. The *ideal* here includes a variant and phonetic frame at the extreme edges of both distributions. Therefore, recognition of these *ideals* may not emerge from the activation of the highly frequent episodes. Yet they are recognized on par with typical forms.

The cluster representations in **Figure 1C** offer a way to think about recognition equivalence in terms of activated episodic clusters and memory inequality in terms of initial encoding strength. The cluster boundaries are used to visualize representational robustness. Here, we have two equally robust forms ([fl
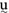

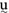
], [flu:t]) and one less robust form ([fl
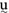

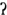
). The shapes correspond to tokens with different pronunciation variants. And, the number of shapes corresponds to frequency. These episodes are encoded at different strengths (visualized as different token sizes), and two clusters that are quantitatively different are predicted to be equally accessible, depending on encoding strength. In the case of [fl
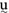

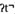
], analogous to a typical female voice, this cluster is composed of a large number of weakly encoded word forms (see also Kuhl, 1991 and Nygaard et al., 2000). In this case, robust activation results from the activation of a densely populated cluster. In the case of the infrequent, non-ideal [flu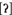] or an atypical, non-socially salient female voice, the cluster is less robust. It contrasts with the typical forms most visibly by quantity. These atypical non-idealized forms are hypothesized to be weakly encoded, like the typical forms. This leads to a less robust cluster, but their acoustic similarities with the typical form leads to the appearance of robustness in immediate processing tasks. Our representation of the infrequent, but idealized, form (C, [flu:t]), illustrates the clusters that result from what we call socially weighted encoding. In this case, we propose that clusters that correspond to atypical, socially idealized forms are as robust as clusters that correspond to typical, non-idealized forms, despite being composed of fewer episodes. In our approach, these episodes are encoded more strongly than their counterparts, as they are experienced in socially salient contexts. Stronger encoding leads to increased specificity and strong verbatim traces (see Brainerd and Reyna, 2002; Brainerd et al., 2008 for supporting arguments). Maintaining the analogy with typical and atypical female voices, we would expect that less typical, but socially salient, female voices should benefit from the same type of encoding benefit. This perspective on representations builds on Johnson’s (2006) account of acoustic-based resonance, but adds a layer of encoding complexity.

The examples provided in **Figure 1** include, along with the frequent, default cluster, two clusters of atypical forms: one that schematically overlaps with the frequent default cluster and another that does not. It is difficult to investigate differences in the encoding of these idealized and non-idealized atypical forms when the non-idealized form, like [fl
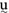

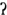
], overlaps with the frequent, default cluster in ways that might boost its activation. To observe the proposed encoding differences, we need an example with a three-way split in these forms in which the default does not overlap with either atypical form. Sumner and Kataoka (2013) provides just such a case, as described before (§ 3). They investigated the effects of talker-specific variation on semantic encoding. The three talkers – an AE, a BE, and a NYC talker– produced forms that are typical for AE listeners (AE talker) or atypical (BE and NYC talkers). The two atypical productions differ from typical AE productions (hence no overlap with the default form) but crucially also differ from each other in perceived standardness. A non-rhotic variant produced by a BE talker is perceived as standard whereas the same variant produced by a NYC talker is perceived as non-standard. Across two experiments, Sumner and Kataoka (2013) found evidence to suggest that stronger encoding of the words uttered by the BE talker leads to recognition equivalence between the AE and BE forms. Importantly, there is a cost found only for the NYC accent, and this, in our view, is partly because these atypical, non-idealized NYC forms do not benefit from acoustic overlap with the AE forms – unlike the atypical, non-idealized examples in **Figure 1**. In some sense, this implies a benefit in the lexical access process for strongly encoded forms, and the equivalence we see results from the power and flexibility stemming from a dense, default cluster (AE) and the increased attention allotted to sparse but idealized productions (BE). Only by including non-overlapping out-of-accent non-idealized productions (NYC) can this benefit be revealed. This claim is consistent with other work showing a benefit in lexical access from increased attention (e.g., Dupoux et al., 2003).

## ACHIEVING SOCIALLY WEIGHTED ENCODING: A DUAL-ROUTE APPROACH

We have suggested that the advancement of theory would benefit from the consideration of the role of social meaning in spoken language understanding. We hypothesize that words are socially weighted. Here, we sketch a way that socially weighted encoding might be accomplished. We suggest that phonetically cued social information is extracted from speech along with linguistic information. And, that this social information modulates the encoding of spoken words and word forms. The general approach and corresponding predictions are outlined below.

### THE SOCIOACOUSTIC AND LINGUISTIC ENCODING OF SPEECH

**FIGURE 2:**
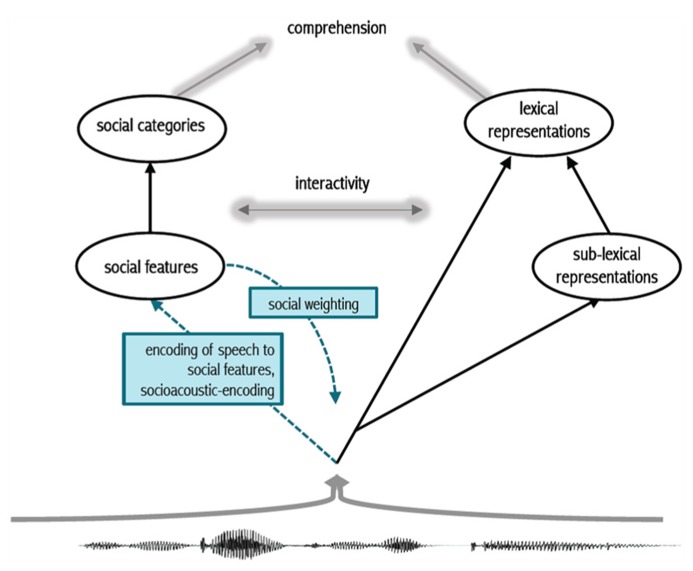
**Schematic of proposed dual-route approach.** In tandem with the encoding of speech to sounds and words (right), acoustic patterns in speech are encoded to social representations (left). Socially weighted encoding results from the heightened activation of social representations that modulates attention to the speech signal. This in turn results in the deep encoding of socially salient acoustic patterns along with linguistic representations, but also independent of them.

We propose that learned acoustic patterns are mapped simultaneously to linguistic representations and to social representations (see Creel and Bregman, 2011 and McLennan and Luce, 2005, for related perspectives). As we describe, one consequence of this dual-route approach is the *socially weighted* encoding of spoken words. The approach depends on resonant activation that modulates attention to speech events by a particular talker or in a particular context. By mapping speech simultaneously to linguistic and social representations, we can arrive at the cluster representations illustrated in **Figure 1**. **Figure 2** illustrates our approach.

In **Figure 2**, listeners map an incoming speech signal to lexical representations either directly, or via smaller linguistic units of representation (see Listener sensitivity to phonetic variation during perception and recognition). This route is represented on the right side of **Figure 2**. We propose an additional encoding route. Building on the finding by Kaganovich et al. (2006) that listeners process pitch simultaneously as linguistic pitch and a cue to speaker gender, we propose that this multiple mapping is a general characteristic of speech processing. The left side of **Figure 2** includes lower-level social features and higher-level social categories. This split is less relevant to the current discussion, but is central to work in other disciplines (see Freeman and Ambady, 2011 for such a split in a theory of persona construal).

We view variation in speech as a social prompt, similar to the visual prompt used in other work (see Listener sensitivity to phonetic variation during perception and recognition). Learned, and subsequently stored, acoustic patterns are associated with social features (e.g., expanded vowel space, slow speech rate and other co-varying patterns may map to the features “formal” or “standard”; a different pattern may map to the feature “female”, while others may map to “foreign”, just as long-lag voice onset time (VOT) and other co-varying patterns map to voiceless word-initial stops in AE). This route helps tease apart productive social sound patterns from lexical representations (e.g., speakers can easily produce non-words or nonsense strings in a carefully- or casually articulated speech style, but we have little understanding of how or why this occurs). We call this new route *socioacoustic-encoding*, indicated in blue on the left side of **Figure 2**. Listeners simultaneously extract linguistic and social information from speech. The activation of salient social features and/or categories induces strong encoding (via increased attention) in a resonant activation network (see Grossberg, 2013 and Kumaran and McClelland, 2012 for the specific dynamics a resonant network). Atypical idealized forms, or atypical socially salient tokens, are predicted to induce greater attention at encoding. This results in a special status for variants and forms that are idealized compared to default variants and forms. A single experience with an ideal variant or form carries more weight than a single experience with a typical non-ideal variant or form. This social weighting has implications for theories of lexical access broadly.

Social weighting also adds a *qualitative* component to the encoding of spoken words.^4^ As listeners, we encounter innumerable instances of a single word. Occasionally, attention is drawn to the specific form of a word. For example, a mother might slow down and produce the rare [t] in the word *city* to aid a child in spelling. In this narrow example, attention is specifically drawn to the *form of the word* (a situation that is extremely rare; any instructor of introductory phonetics can comment on the shock of students when they find out how many acoustic correlates exist for the letter <t> in English). Over the years, orthography, meta-linguistic commentary about standards, and other types of experience (e.g., a [t] along with a careful speech style might be used in an attempt to disambiguate words, or in more formal contexts) compound to contribute to socially salient patterns. Tokens that are congruent with this social salience are more strongly encoded than those heard in default social contexts or than those that are subjectively perceived as defaults.

The result of social weighting is a sparse, high-resolution episodic cluster, which is as easily accessible during lexical access as a dense, low-resolution cluster (see **Figure 1**, bottom). Lexical access is therefore comparable for both clusters, predicting recognition equivalence in a straightforward way. To explain the effects of memory inequality, it is important to understand the effects of encoding strength during presentation at study. Otgaar et al. (2012) have found that attention modulates the ways in which words are encoded. Specifically, they found that words are remembered better in full-attention conditions than in divided-attention conditions. The reason for this improved memory is that full-attention results in greater verbatim encoding as opposed to gist encoding (see also Brainerd and Reyna, 1998). In other words, in a full-attention condition, listeners are more likely to remember exactly what was said, but in a divided attention condition, listeners are better at remembering the general idea of what was said. Along similar lines, the relative contribution of signal-based and knowledge-based information may vary for different speech styles during speech processing (Sumner, 2013). Thus, memory inequality results, in part, from the stronger initial encoding of these atypical idealized forms at study.

### GENERAL PREDICTIONS

Support for this second socioacoustic route will come in various forms. First, we generally expect to find evidence of dual processing. It may well be that the focus on short words and lexical specificity in the field has masked a number of potential effects of socioacoustic encoding. We would expect effects of a dual-route approach in immediate recognition tasks to be most robust in longer utterances, at the ends of experiments, or in words that slow linguistic processing (like words with late disambiguation points). Using longer utterances, Van Berkum et al. (2008) found that listeners’ word predictions in a sentence completion task depended upon the identity of the speaker. And, Creel (2012) found that learned talker-specific color preferences guided children’s behavior in sentence processing. Consistent with the prediction about slowed processing, McLennan and Luce (2005) found stronger talker-based effects in difficult tasks than in easy tasks. Second, we predict recognition equivalence, should we replicate a number of the studies that investigated pronunciation variants outside of a congruent phonetic frame. This would dispense with the representation paradox and provide greater evidence that variants and word forms that are produced at vastly different rates are understood equally well by listeners. Though, as in Johnson (2006), token frequency differences between atypical non-idealized forms and typical non-idealized forms are likely to emerge in psychoacoustic tasks (see **Figure 1A**). Third, listeners should have better memory for atypical idealized forms than for default forms, but also for *subjectively perceived idealized forms*. In other words, memory inequality for socially salient versus default contexts should result from longer-term investigations of variation (where contexts include different speech styles of a single voice, or different voices that are perceived quite differently along social scales by our listener populations). A final prediction, in need of more foundational work, is that individual differences in listeners’ social perceptions of talkers result in memory inequality that depends on these perceptions. These types of effects are likely to be amplified in designs that specifically contrast talkers, making a particular voice socially contrastive with a different voice. In other words, we expect that listeners attend differently to different voices depending on individual-level social perceptions. Here, we provide several more explicit ways some of these predictions can be tested.

### Predictions of the social weighting of spoken words

In terms of social encoding resulting in memory inequality, a number of interesting predictions can be made. First, atypical idealized word forms should be remembered better than typical non-idealized forms. While this has been shown a number of critical comparisons have not yet been made. For example, extending Sumner and Samuel’s (2005) finding that word forms with final [t] are remembered better than forms with the more frequent variants, we would predict this asymmetry to occur more generally across speech styles: words uttered in a careful speech style should be remembered better than words uttered in a casual speech style. Extending this to accents, we might investigate speakers that are generally viewed as prestigious (as prestige has been argued to garner attention; see Chudek et al., 2012) compared to those that are viewed as non-standard. In implicit and explicit memory tasks, then, we would expect stronger encoding for prestigious accents, resulting in better memory compared to that for non-standard speakers. A frequency-driven approach would find it difficult to reconcile this split.

We also expect to see a more pervasive role of phonetically cued social variation in memory-related tasks. For example, Sumner and Samuel (2009) suggested that the memory benefit for rhotic -*er* final forms that resulted independent of listener populations was due in some part to qualitative experience with standard forms. In this case, we might predict that the lower accuracy for non-rhotic items is not because the non-rhotic variant is inherently difficult to remember, but because the non-rhotic variant is produced by a non-standard NYC speaker and the tokens are subsequently weakly encoded. The strongest test of this claim is that the same non-rhotic variant embedded in a prestigious voice (e.g., a prestige accent of BE that is also non-rhotic) would exhibit the memory benefit. This is the exact pattern found by Sumner and Kataoka (2013). In general, the combination of a pronunciation variant *and* a phonetic frame result in social weighting. Extending this line of investigation to other experimental paradigms that are sensitive to encoding differences (like the false memory paradigm, see Gallo, 2006 for a review) should prove worthwhile in understanding effects of social weighting.

### Predictions of a socioacoustic encoding approach

Many of the predictions discussed in this section fall out naturally from models of semantic association where association increases as items or concepts are simultaneously active (see Raaijmakers and Shiffrin, 1980; Landauer and Dumais, 1997; Griffiths et al., 2007). We focus here on predictions that would (1) test the hypothesis that social meaning inferred from phonetic variation in speech occurs independent of the lexicon, and (2) show interactive effects between linguistic and social information. To best illustrate the predicted dissociation between linguistic and socioacoustic encoding, we shift our attention to emotion and gender, though similar predictions extend to careful versus casual speech styles. One prediction we make is that words uttered in a non-neutral intonation should activate words related in meaning to the lexical item *and* to corresponding emotions. Part of this prediction has received some support. Nygaard et al. (2009) investigated the effects of sound symbolism via tone of voice. They recorded six non-words (e.g., *blicket*) with a particular tone of voice to convey happy/sad, short/tall, and other contrastive meanings. In their first experiment, participants heard “Can you get the (happy voiced) blicket one?” and were asked to select either a happy picture or a sad picture. Listeners picked the happy picture more often with happy-voiced *blicket*, and the sad picture more often with sad-voiced *blicket*. While this study shows that listeners use emotional prosody in word learning, the task and the use of non-words limit our understanding of the simultaneous processing of linguistic and social information. The two can be teased apart by investigating the recognition of non-emotion words (e.g., *pineapple*) uttered in a neutral or emotional voice. We predict, for example, that the word *pineapple* uttered in an angry voice should facilitate recognition to the words *fruit* and *upset* in a priming paradigm. In a single-route linguistic encoding approach, a word like *pineapple* uttered in an angry voice should slow lexical access (as atypical utterances are slowed generally). Slowed access should preclude semantic priming (see van Orden and Goldinger, 1994) and, likewise, there should be no priming for emotionally related words. This is one explicit divergence between the current proposal and frequency-dependent approaches to lexical access.

More generally, we predict that words in which the linguistic and socioacoustic cues conflict should result in slowed recognition (Quené et al., 2012). For example, the word *smiling* uttered in an angry voice should be recognized more slowly than the word *smiling* uttered in a happy voice (c.f., Nygaard and Queen, 2008). While **both** are atypical with respect to a listener’s global experience with the word *smiling* and should therefore be slower compared to a neutral control in current theory, the latter benefits from dual encoding. We must also predict, then, that the word *smiling* uttered in a happy voice is recognized more quickly than the word *smiling* uttered in a neutral voice (c.f., Schirmer et al., 2005a,b).

In addition to within talker differences like emotion or speech style, cross-talker differences may also provide support for the dual-route approach. For example, female voices are predicted to activate words associated with the social category of *female*/*woman*. In offline tasks, like a free association task, we might expect that top semantic associates vary by talker gender. We do not predict every word to be associated with a particular gender, nor do we predict differences for every word. Rather, we suggest that, given two different voices, the composition of the top associates across a number of words (typical of free association tasks) will be gender-dependent. These effects should also be observable in online studies. As semantic priming is highly dependent on the association strength between prime and top associate target, we predict that top associate targets obtained from a female voice should be recognized faster when the prime is produced in a female voice than when the prime is produced in a male voice.

In line with the speeded congruency effect discussed, we expect that words associated with a particular gender will be recognized more quickly when that word is spoken by the associated gender than by the non-associated gender. Crucially, this effect should be *independent* of gendered-usage frequency counts. Episodic lexical access models depend heavily on the raw frequency of a particular word uttered by a particular speaker or speaker group (see Walker and Hay, 2011). This approach predicts that words typically uttered by women, for example, are recognized more quickly when produced by women than when produced by men. Our approach suggests that, in addition, gender conceptualization will be a strong predictor of word recognition, independent of whether a particular gender actually utters a gender-associated word more than the other gender. We also predict this effect to bias speech processing early. For example, using the visual world paradigm (see Huettig et al., 2011 for a review), words that are socially associated with a voice (similar to semantic competition effects shown by Huettig and Altmann, 2005) should compete with targets uttered in that voice, but should not compete with targets uttered in different voices. These are a sample of the types of predictions that illustrate the ways in which speech may be encoded simultaneously to social and linguistic representations.

### BROADER IMPLICATIONS

The implications of our approach extend to language processing more generally and might prompt us to question phonetically cued social effects in other domains. Particularly relevant to our discussion is the claim that certain speakers are viewed as unreliable because they are difficult to understand (Lev-Ari and Keysar, 2010). Investigating the effects of comprehension on perceived reliability, Lev-Ari and Keysar (2010) collected comprehension ratings and reliability ratings from native English listeners presented with speech from native and non-native English speakers. Across two experiments, they found that listeners reliably rated non-native English speakers as less reliable than native English speakers. And, the non-native speakers were also rated as more difficult to understand than native English speakers. They claimed that speakers that are difficult to understand are deemed unreliable. The approach they take is [speech] → [linguistic comprehension] → [social judgment]. Our perspective provides an alternate account for these data. In our view, comprehension is the composite of social and linguistic activation. And, phonetic variation that cues unreliability (such as foreign-accents to the ears of many AE listeners) may alter the way one attends to the stimuli (see also Dixon et al., 2002; Gluszek and Dovidio, 2010). It is the age-old question of the chicken and the egg, except that our experimental predictions diverge and the issue can be resolved. The non-native speakers in Lev-Ari and Keysar’s (2010) study were all non-standard, non-prestige speakers and the comprehension scores were based on *perceived* comprehension measures provided by listeners. In a strong post-comprehension social judgment approach, one would expect any accent that is more difficult to understand than a native accent to result in lower reliability ratings. In a strong socioacoustic encoding approach, one would expect that a prestige accent (like a prestige accent of BE) would result in higher ratings of reliability than the native AE accent despite having a different vowel system (see Roach, 2004). Or, that two native AE talkers that are viewed differently along social scales, but are equally easy to understand, should prompt very different reliability ratings. In both cases, collecting both subjective and objective comprehension ratings would be worthwhile, as it is not difficult to imagine a situation in which objective comprehension is the same but subjective comprehension differs. We use this example only to highlight the ways in which we might investigate phonetically cued social effects in spoken word recognition and language comprehension more broadly.

## CONCLUDING REMARKS

In this paper, we have illuminated a growing body of research with data that are not easily accounted for given current theoretical approaches to the perception and recognition of spoken words. We have argued that frequency-based approaches will stall trying to explain recognition equivalence and memory inequality; as will more abstractionist approaches. In order to fully account for the data, we argued that listeners use variation in speech for all of its potential – mapping speech onto linguistic and social representations in tandem. In doing so, we contribute a qualitative component to the definition of listener experience. Our approach raises a number of questions that are beyond the scope of this paper. But, we provided a conceptually feasible approach to the effects of phonetically cued social meaning on cluster representations and speech perception that represents, we believe, a significant departure from previous conceptualizations of the role of variation. We believe that this approach will enable future endeavors to address these questions. Investigating the extensive influence of phonetic variation broadly in speech perception and spoken word recognition should bring us closer to understanding how listeners understand spoken words as produced by a diverse set of speakers.

## Conflict of Interest Statement

The authors declare that the research was conducted in the absence of any commercial or financial relationships that could be construed as a potential conflict of interest.

## Footnotes

1 We use the term idealized here and throughout to refer to a variant or talker that is subjectively viewed as more standard compared to other variants or talkers for a given example (see Campbell-Kibler, 2006 and Sclafani, 2009 for related discussion and references therein).

2 We specifically avoid the term “deleted” as a potentially misleadingly categorical description of a gradient process. See Temple (2009) for further discussion.

3 We highlight here instances in which equivalence across variants is established. We do not claim that all variants (in all contexts across all talkers and accents) should result in equivalence. See Sumner and Kataoka, 2013 for a related discussion about the tension between factors such as exposure frequency and language ideologies.

4 While social categories themselves can be understood in quantitative measures, qualitative in the present context refers only to the influence on social meaning on processes and representations involved in spoken language understanding.
